# The emerging role of a Stroke Clinical Nurse Specialist (CNS) in Early Supported Discharged: Developing a pathway for stroke nursing for secondary prevention in the community.  A scoping review protocol.

**DOI:** 10.12688/hrbopenres.13818.1

**Published:** 2024-01-08

**Authors:** Sarah-Jane Byrne, David J. Williams, Declan Patton, Paul J. Murphy, Frances Horgan

**Affiliations:** 1 School of Postgraduate Studies, Royal College of Surgeons in Ireland, RCSI, University of Medicine and Health Sciences, Dublin 2, Ireland; 2 Department of Geriatric and Stroke Medicine, Beaumont Hospital, Dublin 9, Ireland; 3 Department of Geriatric and Stroke Medicine, Royal College of Surgeons in Ireland, RCSI, University of Medicine and Health Sciences, Dublin, Dublin 2, Ireland; 4 RCSI School of Nursing and Midwifery, Royal College of Surgeons in Ireland, RCSI, University of Medicine and Health Sciences, Dublin 2, Ireland; 5 RCSI Library, Royal College of Surgeons in Ireland, RCSI, University of Medicine and Health Sciences, Dublin 2, Ireland; 6 RCSI, School of Physiotherapy, Royal College of Surgeons in Ireland, RCSI, University of Medicine and Health Sciences, Dublin, Dublin 2, Ireland

**Keywords:** Early Supported Discharge, Stroke Clinical Nurse Specialist, Stroke Rehabilitation, Community Care, Primary Care, Scoping Review

## Abstract

**Background:**

Stroke represents a major source of mortality and morbidity globally. The role of a stroke Clinical Nurse Specialist (CNS) as an expert team member in early supported discharge (ESD) for stroke, is not well defined or described although it is well established in other models of after-hospital and out-reach specialist care in the community. A greater focus has been on patients receiving rehabilitation post-stroke, however there is a need for a more holistic approach to care which clinical nurse specialists can offer to patients as part of ESD. Nurses are often the cohesive point of contact for other after-hospital services, managing many aspects of secondary prevention.

**Objective:**

The aim of this scoping review is to explore the evidence in relation to the role of the stroke nurse providing secondary prevention interventions to stroke patients in a community setting.

**Methods:**

We will conduct a scoping review in accordance with the Arksey and O’Malley, 2005 ^1^ scoping review framework and the PRISMA-ScR guidelines to map available literature on the role of the stroke nurse in post-stroke care of patients in the community. The Cochrane Central Register of Controlled Trials and Systematic literature searches including databases MEDLINE, EMBASE, CINAHL, google scholar and grey literature will be searched using keyword searches. Data will be charted and synthesised and a narrative synthesis will be conducted.

**Conclusions:**

This scoping review will be used to identify gaps in the current literature and identify areas for future research in the role of the stroke nurse in ESD in relation to secondary prevention for stroke patients and inform the development of a pathway for stroke nursing in ESD.

## Introduction

Stroke represents a major source of mortality and morbidity globally, and it is reported that there are over 15 million individuals affected worldwide by stroke every year ^2, 3^. Nurses play a critical role in all aspects of stroke patients care including initial assessment, diagnosis and provision of inpatient post-stroke secondary prevention care until the patient is discharged home ^4, 5^. Less attention has been given to the post-stroke role of the clinical nurse specialist as part of an early supported discharge (ESD) model of care ^4^. It is recommended in the National Stroke Strategy 2022–2027 ^6^ that a stroke nurse is part of the composition of ESD Teams, however this is not the case in all hospital models of ESD teams. The role of a specialist nurse in ESD is still poorly described though well established in similar models of chronic care in community settings. There is a wealth of information in the literature regarding stroke nurses delivering secondary stroke prevention education to patients and the stroke care pathway, including secondary prevention is discussed as being a comprehensive and personalised approach in the National Institute for Health and Care Excellence Guidelines ^7^ and ISWP define abbreviation guidelines ^8^.

The role of a Stroke Clinical Nurse Specialist as an expert team member in ESD for stroke, is not well defined or described although it is well established in other models of after-hospital and out-reach specialist care in the community such as palliative, respiratory and cardiac care ^4^. In their meta-analysis of eleven randomised control trials in six countries, examining the composition of the ESD teams, Langhorne and Baylan found no significant involvement of nurses, within ESD teams ^5, 9^. Nurses are often the cohesive point of contact for other after-hospital services, managing many of the aspects of secondary prevention, psychological and functional problems and patient education in models of chronic disease management ^10, 11^. While there has been a focus on patients receiving rehabilitation post-stroke, the role of nurse specialists and what they can offer to patients as part of ESD has received little attention. Post-stroke nursing interventions are critical in not only the initial inpatient setting, but also the home and community setting ^4^. As part of an ESD team, the rehabilitation therapists have an important role in improving sensory and motor impairments and functional outcomes, while, stroke clinical nurse specialists play an important role in assisting the patient reducing co-morbid conditions through secondary prevention (e.g., hypertension, smoking and alcohol cessation and medication management) thus limiting the likelihood of recurrent stroke and assisting the patient in their adjustment to lifestyle ^5^.

## Methods

### Study design

This scoping review will be valuable in identifying a gap in the literature, scope a body of literature and clarify the role of the stroke nurse in ESD. This could in future potentially be used a precursor for more focused systematic reviews and/or meta-analyses. The scoping review framework by Arksey and O’Malley ^1^ will be used. This framework outlines five steps for a rigorous scoping review: (1) identifying the research questions; (2) searching for relevant studies; (3) selecting studies; (4) charting the data, and; (5) collating, summarising, analysing and presenting the results. This framework highlights that while carrying out a scoping review, rigorous and transparent methods are upheld to ensure the results are reliable ^12^. The Preferred Reporting Items for Systematic reviews and Meta-Analyses (PRISMA) extension for Scoping Reviews (PRISMA-ScR) checklist will be used to guide the reporting of this review ^13^. This protocol is reported in line with the PRISMA-P checklist ^13^.

## Stage 1: Identifying the research question

Central Research Question: What is the emerging role of Stroke Clinical Nurse Specialist in ESD: informing a pathway and model of care for stroke patient secondary prevention in the community.

### Eligibility criteria

- Research studies with any intervention that provides community-based care (intervention/strategies/programmes for stroke recovery after discharge from an acute care or rehabilitation hospital) and delivered by nurses in home/community settings.
- Studies with an intervention that focused on stroke survivors after discharge from an acute care or rehabilitation hospital.

### Exclusion criteria

- Research studies with no stroke specific interventions.
- Studies with no nursing involvement.

## Stage 2: Identifying relevant studies

A library will be created on EndNote X9 for this scoping review. In conjunction with an information specialist librarian, a comprehensive search strategy will be outlined and conducted to identify the relevant literature in relation to the research question. The Cochrane Central Register of Controlled Trials and Systematic literature searches including databases MEDLINE, EMBASE, CINAHL, google scholar and grey literature will be searched using keyword searches. We will screen the reference lists of the selected articles for inclusion to identify any potential additional appropriate studies. We undertook a preliminary search in October 2022 using search keywords (See Table 1). Following this, the search strategy will be adapted and a new search will be conducted in conjunction with an information specialist librarian (PM).

**Table 1.** Library Search.

	OVID MEDLINE All	xxxxxxxxx
1	exp Stroke/OR exp Stroke Rehabilitation/OR (stroke adj2 rehabilitation) OR (stroke adj2 recovery) OR (post adj2 stroke) OR *Cerebrovascular Disorders/	206877
2	exp Nursing/OR (nurse OR nurses OR nursing).mp.	782698
3	Exp Community Health Services/OR exp Patient Discharge/OR (patient$ adj2 discharge) OR ((community adj2 care) OR (home adj2 care) OR (continuity adj2 care) OR (transitional adj2 care) OR (transition adj2 care) OR (secondary adj2 prevention)).mp.	449430
4	1 AND 2 AND 3	1123
5	Limit 4 to English language	990
	**EMBASE (Elsevier.com)**	
1	'cerebrovascular accident'/exp OR stroke:ti,ab,de OR (stroke NEXT/2 rehabilitation) OR (stroke NEXT/2 recovery) OR (post NEXT/2 stroke)	599,093
2	'nursing'/exp OR 'nurse'/exp OR nurse$:ti,ab OR nursing:ti,ab	831,763
3	'community care'/exp OR (community NEXT/2 care):ti,ab,kw OR 'hospital discharge'/exp OR ((patient NEXT/2 discharge) OR (home NEXT/2 care) OR (continuity NEXT/2 care) OR (transitional NEXT/2 care) OR (transition NEXT/2 care) OR (secondary NEXT/2 prevention)):ti,ab,kw	393,002
4	1 AND 2 AND 3	2,192
5	Limit 4 to English language articles AND Embase records only	663
	**CINAHL on Ebscohost**	
1	(MH "Stroke+") OR (MM "Stroke Patients") OR TX ((stroke N2 rehabilitation) OR (stroke N2 recovery) OR (post N2 stroke) OR (Cerebrovascular N2 Disorder$))	97,572
2	(MH "Community Health Nursing+") OR TI (nurse OR nurses OR nursing) OR AB (nurse OR nurses OR nursing) OR SU (nurse OR nurses OR nursing)	997,039
3	(MH "Early Patient Discharge") OR (MH "Patient Discharge") OR TX ((hospital N2 discharge) OR (community N2 care) OR (home N2 care) OR (transitional N2 care) OR (transition N2 care) OR (secondary N2 prevention))	239,023
4	1 AND 2 AND 3	1,339
5	Limit 5 to English language AND journal articles	878

## Stage 3: Selecting studies

Following the search to identify any relevant studies, the final included studies will be imported to ENDNote X9 and the duplicates will be removed where possible. The primary researcher (SJB) will carry out an initial scan of the literature to evaluate and exclude irrelevant literature as specified in the eligibility criteria. Two reviewers (SJB and FH) will independently review the remaining titles and abstracts and apply the inclusion criteria to all remaining studies. A third and fourth reviewer (DP and DW) will act as arbitrator in the event of any disagreements. The two reviewers (SJB and FH) will meet to reach consensus regarding the full text inclusion and these will be uploaded onto the EndNote X9 library. Studies that do not meet the inclusion criteria will be excluded. Reasons for the exclusion will be kept and presented as part of the flow diagram. The final search results will be outlined in a PRISMA flow diagram from the PRISMA-ScR statement, which will be accompanied by a narrative description of the process.

## Stage 4: Charting the data

This scoping review is designed to identify the range of evidence available in the literature which will be represented as a mapping of the identified data without the act of synthesis or referring to any particular methodological qualities of the studies chosen. All relevant data will be extracted from each included study to inform the scoping review objectives and questions. Key information regarding the role of the stroke clinical nurse specialist in secondary stroke prevention in the community will be organised into categories. A narrative synthesis of the literature and how it is applicable to the role of the stroke nurse in early ESD will be conducted. As part of this process, one reviewer (SJB) will independently chart the data from the retrieved articles via a standardised form created using Microsoft Excel software developed from the JBI extraction tool ^12, 14^. The second reviewer (FH) will check a sample of 20% of the charted data. They will then discuss the results and update the charting form in an iterative process. Reasons for changes will be outlined and presented as an appendix as part of the review. If there are any inconsistencies these will be reviewed by a third reviewer and fourth reviewer (DW and DP).

## Stage 5: Collating, summarising, analysing and presenting the results

Results will be reported using the PRISMA-ScR guidelines ^15^. The research question will be reported as narrative summary. This narrative description will be used to synthesise the study findings based on the themes generated from the extracted data.

## Dissemination

The findings of the scoping review will be published in a peer-reviewed open-source journal; presented at national and international conferences; and shared with researchers, clinicians, stroke survivors and families through organisations for people with stroke.

## Ethics

This scoping review consists of collecting, collating, summarising, analysing and presenting a narrative synthesis of the data from publicly available material and therefore does not require ethics approval. This scoping review constitutes the first step in a multi-phased research project aimed at developing a pathway for stroke nursing in ESD and a secondary prevention model of care for patients post stroke. The results from this scoping review will guide and be combined with data from later phases of the research, including surveys with stroke survivors and healthcare professionals, and a co-design process. Ethics approval will be sought for these later stages of the research.

## Discussion

The proposed scoping review will focus on how nurses in community settings as part of ESD can play a role in delivering a personalised and comprehensive stroke secondary prevention education to patients and the potential benefits to patients. This scoping review will highlight the role of community rehabilitation nurses on the ESD team providing their stroke rehabilitation skills and experience, thereby strengthening the model of interdisciplinary teamworking ^10, 16, 17^. The contribution of nursing to an ESD team enables the stroke patient to be cared for holistically, focusing on multiple domains of care that are individual to the patient. Furthermore, the results of this scoping review will guide any future research in the novel role of the stroke nurse in early supported discharge.

## Data Availability

No data is associated with this article.
