# Supplementary material for: The emerging role of a Stroke Clinical Nurse Specialist (CNS) in Early Supported Discharged: Developing a pathway for stroke nursing for secondary prevention in the community.  A scoping review protocol
Source: HRB Open Res. 2024 Oct 7;7:2. Originally published 2024 Jan 8. [Version 2] doi: 10.12688/hrbopenres.13818.2 (PMC11544196; doi:10.12688/hrbopenres.13818.2)
Supplement: Peer review history

# Reviewer report: Reviewer response for version 2

**DOI:** 10.21956/hrbopenres.15354.r42649

**Published:** 2024-11-07

**Authors:** Ashan Weerakkody

**Affiliations:** 1 School of Medical and Health Sciences, Edith Cowan University, Joondalup, Western Australia, Australia

Dear authors,

 Thank you once again for the opportunity to review this protocol. I think this is novel and important work, so I wish you the best in your course of research- which this appears to be the commencement of a larger project. 

 Introduction:

 Your amendments now more clearly outline the aims and justification for the scoping review. 

 Methods:

 Eligibility clearer, and clearly justifies why increasing search to community/post-discharge in lieu of anticipated limited research into ESD. 

 Discussion:

 This is a much better summary of the potential contributions to future work and understanding that can arise from this review.

Is the study design appropriate for the research question?

Yes

Is the rationale for, and objectives of, the study clearly described?

Partly

Are sufficient details of the methods provided to allow replication by others?

Partly

Are the datasets clearly presented in a useable and accessible format?

Not applicable

Reviewer Expertise:

Stroke rehabilitation, early-supported discharge, neurological physiotherapy, qualitative research, implementation science.

I confirm that I have read this submission and believe that I have an appropriate level of expertise to confirm that it is of an acceptable scientific standard.

# Reviewer report: Reviewer response for version 2

**DOI:** 10.21956/hrbopenres.15354.r42650

**Published:** 2024-10-14

**Authors:** Rita Chiaramonte

**Affiliations:** 1 University of Catania, Catania, Italy

This is a very interesting scoping review on the role of stroke nurses in providing secondary prevention interventions to stroke patients in a community setting.

 Here my suggestions:

**Keywords**: Prefer Mesh Keywords

**Methods**: The bullet points are clear, but bullet points are not typically used in the main text. I suggest making the content flow more smoothly within the text. If desired, you could add a table to present the inclusion and exclusion criteria in a more structured way.

**Results**: Although this is a protocol, it would be helpful to outline the specific roles of nurses in this field, as they may serve as prognostic indicators for the outcomes you expect to find (such as: 1. **Monitoring and Managing Risk Factor** like hypertension, diabetes, ... **2.** **Patient Education **for prevention strategies, **3. Medication Management** in collaboration with physicians, **4.** **Rehabilitation Support **reinforcing physiotherapy encouraging mobility, speech therapy, and facilitate exercises that help regain independence in ADLs, **5.** **Care Coordination **between patient, family, multidisciplinary team, **6. Infection Prevention and Control** like Cl. diff (Chiaramonte R et al, 2023) [Ref 1], UTIs, Pneumonia, **7. Health Promotion**:​​​​​, **8. Discharge Planning and Transition of Care **including home care instruction infection prevention

Is the study design appropriate for the research question?

Partly

Is the rationale for, and objectives of, the study clearly described?

Partly

Are sufficient details of the methods provided to allow replication by others?

Yes

Are the datasets clearly presented in a useable and accessible format?

Partly

Reviewer Expertise:

Rehabilitation in neurological disorders, swallowing disorders, botulinum toxin injections, scoliosis, imbalance

I confirm that I have read this submission and believe that I have an appropriate level of expertise to confirm that it is of an acceptable scientific standard, however I have significant reservations, as outlined above.

## References

[1] : Impact of Clostridium difficile infection on stroke patients in rehabilitation wards. *Infectious Diseases Now* .2023;53(5) : 10.1016/j.idnow.2023.104685 10.1016/j.idnow.2023.104685 36841431

# Reviewer report: Reviewer response for version 1

**DOI:** 10.21956/hrbopenres.15125.r38346

**Published:** 2024-04-30

**Authors:** Suzanne Hoi Shan Lo

**Affiliations:** 1 Nethersole School of Nursing, Faculty of Medicine, The Chinese University of Hong Kong, Hong Kong, Hong Kong

Thank you for the opportunity to review this manuscript. It is a scoping review protocol and some suggestions for the authors consideration:

 - the eligibility criteria of studies to be included are too broad for example, all interventions delivered by nurse at acute or home settings. It may retrieve many articles and create large variations in the results. Could be more specific by specifying the criteria for early supported discharged, and the role of a stroke clinical nurse to be included. Besides, the topic of the manuscript is for secondary prevention in the community, may also highlight this in the eligibility criteria.

 - the search for the articles could be more extensive by searching for grey literatures, theses and dissertations, ongoing trials or unpublished studies.

Is the study design appropriate for the research question?

Yes

Is the rationale for, and objectives of, the study clearly described?

Partly

Are sufficient details of the methods provided to allow replication by others?

Partly

Are the datasets clearly presented in a useable and accessible format?

Not applicable

Reviewer Expertise:

Stroke care

I confirm that I have read this submission and believe that I have an appropriate level of expertise to confirm that it is of an acceptable scientific standard, however I have significant reservations, as outlined above.

# Response

**Published:** 2024-10-09

**Authors:** Sarah-Jane Byrne

**Affiliations:** Royal College of Surgeons in Ireland, RCSI, University of Medicine and Health Sciences, Ireland

Thank you, Professor, Hoi Shan Lo, for your time in reviewing our protocol. We appreciate all your comments and suggested changes. We have addressed each of the points you have raised and amended the paper accordingly. This is now described below.

**Reviewer comment 1:** The eligibility criteria of studies to be included are too broad:

**Author response:** This area of research is novel as there are extremely limited prior systematic reviews or scoping reviews completed on the role of the stroke nurse as an expert in early supported discharge. The broad eligibility criteria in this scoping review protocol is to highlight that we will review general stroke nursing in a community setting and highlight how the role would be applicable to the stroke nurse specialist on an early supported discharge team and provide the first narrative synthesis of the role of the nurse as an expert in early supported discharge.

**Reviewer comment 2:**. The search for the articles could be more extensive by searching for grey literatures, theses and dissertations, ongoing trials or unpublished studies:

**Author response: **We have included grey literature and any potential additional appropriate studies in our search strategy.

# Reviewer report: Reviewer response for version 1

**DOI:** 10.21956/hrbopenres.15125.r38350

**Published:** 2024-03-27

**Authors:** Rita Chiaramonte

**Affiliations:** 1 University of Catania, Catania, Italy

This study examines the role of the Stroke Clinical Nurse Specialist.

 It is a protocol of a review. This kind of pilot study is usual for a systematic review less for a narrative review.

 However, my suggestions aim to better contextualize the study and make the text more readable for readers that should appreciate to know the key points of the future narrative review.

1. Keywords: I suggest using Mesh keywords.
2. Introduction: At the end of the introduction, please add the aim of the study.
3. Additionally, consider including key points you intend to investigate in the next narrative review, such as:
  - Complications related to bed confinement, including: a) Respiratory failure (Robateau Z, et. al., 2024 [Ref 1]) b) Pressure injury (Schott M, et. al., 2024 [Ref 2]) c) Infection (Chiaramonte R, et. al., 2023 [Ref 3])
  - motion (Chiaramonte R, et. al., 2022 [Ref 4])
  - communication (Chiaramonte R, et. al., 2021 [Ref 5])
  - and so long...
4. You could start to add the importance of a multidisciplinary teams.
5. The suggestions are related to my literature knowledge, I invited the authors to explore other relevant references

Is the study design appropriate for the research question?

Partly

Is the rationale for, and objectives of, the study clearly described?

Partly

Are sufficient details of the methods provided to allow replication by others?

Yes

Are the datasets clearly presented in a useable and accessible format?

Partly

Reviewer Expertise:

Rehabilitation in neurological disorders, swallowing disorders, botulinum toxin injections, scoliosis, imbalance

I confirm that I have read this submission and believe that I have an appropriate level of expertise to confirm that it is of an acceptable scientific standard, however I have significant reservations, as outlined above.

## References

[1] : Acute Respiratory Failure in Severe Acute Brain Injury. *Crit Care Clin* .2024;40(2) : 10.1016/j.ccc.2024.01.006 367-390 10.1016/j.ccc.2024.01.006 38432701

[2] : Nutritional factors and pressure injury risk in hospitalised patients post-stroke. *J Wound Care* .2024;33(Sup2) : 10.12968/jowc.2024.33.Sup2.S32 S32-S39 10.12968/jowc.2024.33.Sup2.S32 38348865

[3] : Impact of Clostridium difficile infection on stroke patients in rehabilitation wards. *Infectious Diseases Now* .2023;53(5) : 10.1016/j.idnow.2023.104685 10.1016/j.idnow.2023.104685 36841431

[4] : Proprioceptive and Dual-Task Training: The Key of Stroke Rehabilitation, A Systematic Review. *J Funct Morphol Kinesiol* .2022;7(3) : 10.3390/jfmk7030053 10.3390/jfmk7030053 35893327 PMC9326539

[5] : A Systematic Review of Measures of Dysarthria Severity in Stroke Patients. *PM R* .2021;13(3) : 10.1002/pmrj.12469 314-324 10.1002/pmrj.12469 32818305

# Response

**Published:** 2024-10-09

**Authors:** Sarah-Jane Byrne

**Affiliations:** Royal College of Surgeons in Ireland, RCSI, University of Medicine and Health Sciences, Ireland

Thank you, Dr Chiaramonte, for your time in reviewing our protocol.

 We appreciate all your comments and suggested changes. We are extremely grateful to you for giving of your time and expertise and hope the attached responses address your concerns and comments. We have addressed each of the points you have raised and amended the paper accordingly. This is now described below.

  

1. Keywords: We will use the following Mesh keywords: Early Supported Discharge, Nurse, Stroke Rehabilitation, Community Health Services, Primary Health Care.
2. Introduction: We have added the aim of the study at the end of the introduction paragraph.
3. Consider including key points you intend to investigate in the next narrative review: We have identified that this scoping review will be valuable in identifying a gap in the literature, scope a body of literature and clarify the role of the stroke nurse in ESD. This could in future potentially be used a precursor for more focused systematic reviews and/or meta-analyses.
4. You could start to add the importance of a multidisciplinary teams: We have added details regarding the importance of multidisciplinary teams in the introduction.

# Reviewer report: Reviewer response for version 1

**DOI:** 10.21956/hrbopenres.15125.r37786

**Published:** 2024-02-16

**Authors:** Ashan Weerakkody

**Affiliations:** 1 School of Medical and Health Sciences, Edith Cowan University, Joondalup, Western Australia, Australia

Thanks for allowing me to review this protocol. The role of stroke clinical nurse specialists is understated, and I commend this research direction your scoping review is taking. 

**Summary:**

 Intro: 

 The authors outline the impact of stroke and the role nurses have traditionally played in initial assessment, diagnosis, provision of secondary stroke prevention education in inpatient settings. They then discuss the limited literature on stroke CNSs working in ESD services despite similar roles in other community multidisciplinary teams. They report potential benefits of including nurses in ESD teams such as a liaison point for other out-of-hospital teams and supporting chronic disease management. 

 Methods: 

 The authors identify the scoping review framework they will employ and outline how they will undertake each of the 5 stages. The methods appear appropriate, however further detail is required to outline aspects of data analysis and synthesis (see below)

 I imagine much of my comments may be fully addressed during the write up for publication when you have your results, however these are questions I had which may be good to address as part of this protocol for clarity to the reader and to better understand the purpose, process and impact of this review. 

 General comments:

 There are some punctuation issues that affect readability, particularly incorrect placement of commas in longer sentences. 

 Intro: 

 It would be good to outline some of the benefits to CNSs being part of other community teams (palliative, respiratory) to strengthen the argument for nurses in ESD teams. Maybe a statement on why nursing in ESD may be superior to only in inpatient settings if you are able to reference this- it would add strength to the need for this review. Can you add a sentence or two summarising the need for this review at the end of the intro? This would bring the two introduction paragraphs together better. 

 Methods:

 The first 2 sentences outlining the potential value of this review to scope the literature and act as a precursor for future systematic reviews would be better placed at the end of the introduction or under the heading "Aims" in the methods section- as it is not a statement regarding study design. 

 Regarding your study inclusion criteria- you don't mention ESD, only community/home. It may be difficult to generalise your findings from community services to specifically ESD; as while ESD is home/community based, not all home/community teams are ESD. I'm thinking this might be because there is little/no literature on specific ESD so you've expanded to stroke community teams. However, you should probably include 2 questions here- 1 on ESD, and 1 on community stroke teams in lieu of sufficient data for ESD only and a statement to explain this. 

 The authors mention that the study findings will be synthesised from the themes generated. How are you doing this? Are you using a framework such as thematic synthesis or other? Do you anticipate qualitative and/or quantitative studies? Will you synthesise both forms the same way? 

 Ethics

 The statement is long considering this is a review of already published studies. The comments regarding future work, guided by findings from this review would be better placed in the discussion section. 

 Discussion

 This section is too brief. It would be best to include how this review will inform future work here, rather than in the ethics statement. 

 Advice on screening:

 This is more to save you time rather than as part of peer review. Have you considered a semi-automated screening tool to support your abstract and title screening? I've used Research Screener previously, and have included its reference and another systematic review evaluating the performance of these tools.

Is the study design appropriate for the research question?

Yes

Is the rationale for, and objectives of, the study clearly described?

Partly

Are sufficient details of the methods provided to allow replication by others?

Partly

Are the datasets clearly presented in a useable and accessible format?

Not applicable

Reviewer Expertise:

Stroke rehabilitation, early-supported discharge, neurological physiotherapy, qualitative research, implementation science.

I confirm that I have read this submission and believe that I have an appropriate level of expertise to confirm that it is of an acceptable scientific standard, however I have significant reservations, as outlined above.

## References

[1] : Methods for the thematic synthesis of qualitative research in systematic reviews. *BMC Med Res Methodol* .2008;8: 10.1186/1471-2288-8-45 45 10.1186/1471-2288-8-45 18616818 PMC2478656

[2] : Research Screener: a machine learning tool to semi-automate abstract screening for systematic reviews. *Syst Rev* .2021;10(1) : 10.1186/s13643-021-01635-3 93 10.1186/s13643-021-01635-3 33795003 PMC8017894

[3] : Reducing Literature Screening Workload With Machine Learning. *Zeitschrift für Psychologie* .2023;231(1) : 10.1027/2151-2604/a000509 3-15 10.1027/2151-2604/a000509

# Response

**Published:** 2024-10-09

**Authors:** Sarah-Jane Byrne

**Affiliations:** Royal College of Surgeons in Ireland, RCSI, University of Medicine and Health Sciences, Ireland

Thank you, Mr Ashan Weerakkody for your time in reviewing our protocol. Thank you for this positive feedback on our protocol. We appreciate all your comments and suggested changes. We have addressed each of the points you have raised and amended the paper accordingly. This is now described below.

1. We have corrected the grammar and punctuation errors in the protocol.
2. Introduction: We have clarified the aim of the study in the introduction paragraph, and we have added a statement to summarise the need for this review at the end of the introduction section.
3. Methods: thank you for this feedback, we have moved this statement to the end of the introduction.
4. Inclusion Criteria: we have amended the criteria to include on ESD, and community stroke teams.
5. Study Findings: We anticipate both qualitative and quantitative papers. Qualitative data gathered will be presented as a descriptive narrative and it is beyond the remit of a scoping review to perform a thematic analysis.
6. Ethics: We have condensed the ethics paragraph and moved the appropriate section to the discussion section.
7. Discussion: We have further developed the discussion section and added in details regarding how this review will inform future work.
8. We wish to thank you for your valuable advice on the use of a screening tool. We will complete title and abstract screening in the Covidence platform.
